# Role of Long Non-Coding RNAs in Pulmonary Arterial Hypertension

**DOI:** 10.3390/cells10081892

**Published:** 2021-07-26

**Authors:** Yun Han, Md Khadem Ali, Kamal Dua, Edda Spiekerkoetter, Yuqiang Mao

**Affiliations:** 1 Department of Thoracic Surgery, Shengjing Hospital, China Medical University, Shenyang 110022, China; hany_sjhosp@126.com; 2 Division of Pulmonary and Critical Care Medicine, School of Medicine, Stanford University, Stanford, CA 94305, USA; mdali@stanford.edu (M.K.A.); eddas@stanford.edu (E.S.); 3 Vera Moulton Wall Center for Pulmonary Vascular Disease, Stanford University, Stanford, CA 94305, USA; 4 Discipline of Pharmacy, Graduate School of Health, University of Technology Sydney, Sydney, NSW 2007, Australia; Kamal.Dua@uts.edu.au

**Keywords:** pulmonary arterial hypertension, long non-coding RNAs, right ventricle, pulmonary vascular remodeling, cell proliferation

## Abstract

Pulmonary arterial hypertension (PAH) is a debilitating condition of the pulmonary circulatory system that occurs in patients of all ages and if untreated, eventually leads to right heart failure and death. Despite existing medical treatment options that improve survival and quality of life, the disease remains incurable. Thus, there is an urgent need to develop novel therapies to treat this disease. Emerging evidence suggests that long non-coding RNAs (lncRNAs) play critical roles in pulmonary vascular remodeling and PAH. LncRNAs are implicated in pulmonary arterial endothelial dysfunction by modulating endothelial cell proliferation, angiogenesis, endothelial mesenchymal transition, and metabolism. LncRNAs are also involved in inducing different pulmonary arterial vascular smooth muscle cell phenotypes, such as cell proliferation, apoptosis, migration, regulation of the phenotypic switching, and cell cycle. LncRNAs are essential regulators of gene expression that affect various diseases at the chromatin, transcriptional, post-translational, and even post-translational levels. Here, we focus on the role of LncRNAs and their molecular mechanisms in the pathogenesis of PAH. We also discuss the current research challenge and potential biomarker and therapeutic potentials of lncRNAs in PAH.

## 1. Introduction

Pulmonary arterial hypertension (PAH) is a devastating cardio-pulmonary disease characterized by progressive remodeling of the pulmonary vessels that results in a narrowing of the arterial lumen, leading to an increase in pulmonary vascular resistance and elevation of pulmonary arterial pressure and eventually right ventricular (RV) failure and death. Dysfunction of pulmonary vascular cells such as pulmonary arterial endothelial cells (PAECs), pulmonary arterial smooth muscle cells (PASMCs), fibroblasts, dysregulation of the immune system, and impaired angiogenesis are collaboratively involved in promoting pulmonary vascular remodeling [1]. While PAH is a relatively rare disease, affecting about one to two persons per million yearly, the morbidity and mortality rates are high as it leads to RV failure and death within 2–3 years if left untreated [2,3]. The exact cause for PAH is unknown, but it may be associated with genetic (mutations) or epigenetic changes, immune and inflammatory triggers, environmental factors (e.g., hypoxia, viral infections, anorectic agents etc.), and altered metabolism. Our understanding of the molecular and cellular basis for PAH pathogenesis has significantly progressed. While currently available treatment options have been shown to increase survival and improve quality of life, the disease however remains incurable. Therefore, new effective therapies are urgently needed. Understanding the molecular mechanisms that underlie the pathogenesis of PAH is essential in discovering novel treatments for PAH.

Non-coding RNAs (ncRNAs), mostly micro-RNAs, have recently become an area of increasing interest for the role they play in health and disease. Emerging evidence suggests that a subgroup of ncRNAs, long non-coding RNAs (lncRNAs), endogenously expressed RNAs (≥200 nt length) that lack protein coding ability, nevertheless play important roles in a range of pathophysiological processes, such as regulation of cellular activities (e.g., proliferation, migration, apoptosis, angiogenesis, metabolism), inflammatory and immune responses, and vascular angiogenesis [4,5,6,7,8,9,10]. LncRNAs have also been found to play a key regulative role in osteogenesis [11]. Based on their genomic location near protein-coding regions, lncRNAs are classified into five categories: sense, antisense, intronic, intergenic, and bidirectional [12]. Generally, lncRNAs are transcribed by RNA polymerase II and the premature lncRNAs are post transcriptionally modified, such as 5′- ends capped with 7-methyl-guanosine, alternatively spliced, 3′-polyadenylated similar to protein coding mRNAs [13]. In addition, several other pathways contribute to the generation of lncRNAs. For example, RNA polymerase III generates non-polyadenylated lncRNAs, and some lncRNAs are generated during snoRNA production [14]. Compared with mRNAs, lncRNAs expression is highly cell type- and tissue-specific, and they have longer but fewer exons [15]. LncRNAs are usually of low abundance, less evolutionary conserved, mostly localized in the nucleus but also found in the cytoplasm, while their subcellular localization can influence the regulation mechanism and functional models [16,17]. For a large number of lncRNAs their precise function is still unknown, therefore, their exact classification is unclear because of the lack of detailed functional characterization. LncRNAs regulate gene expression at the epigenetic, transcriptional, and post-transcriptional levels via acting as guide, decoy, scaffold, and signaling molecules [18]. LncRNAs can also indirectly regulate gene expression via acting as competing endogenous RNAs, sponging off miRNAs from their mRNA targets [19]. Previous studies suggest that abnormally expressed lncRNAs are strongly linked with the pathogenesis of many human diseases, including cardiovascular disorders [20,21,22], respiratory disease [23], and different types of cancer [24,25].

Cancer and PAH exhibit many similar characteristics, such as sustained cell proliferation and apoptosis resistance, abnormal angiogenesis, and dysregulated cellular metabolism [26]. LncRNAs may regulate cell proliferation, migration, cell cycle, apoptosis, DNA damage, metabolism, and immune function in human cancers through different mechanisms [14,27]. There is evidence that lncRNAs are also involved in the proliferation, apoptosis, and cell cycle of PAECs and PASMCs in PAH [28]. Recently, lncRNAs have also been shown to be implicated in pulmonary vascular remodeling and PAH pathogenesis [29]. In this review, we discuss lncRNAs and the molecular mechanisms of their involvement in the development and progression of PAH. We also highlight the challenges of lncRNAs research and point to potential therapeutic applications as biomarkers and treatments in PAH.

## 2. LncRNAs in PAH

**Figure 1:**
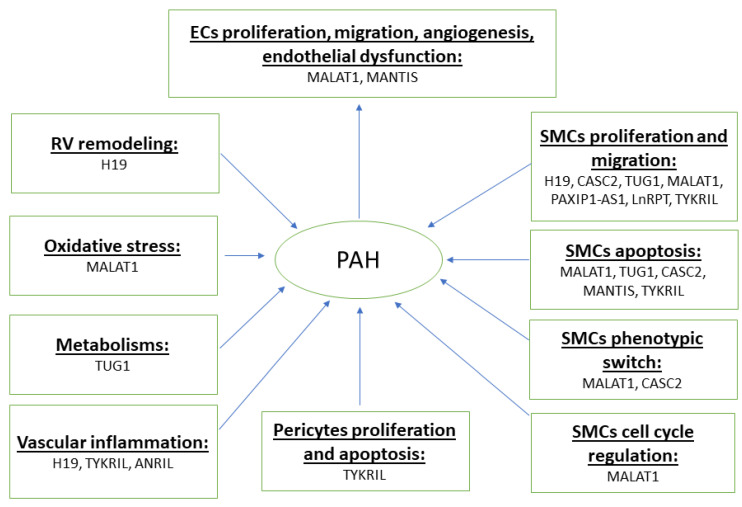
Role of lncRNAs involved in PAH.

Emerging studies suggest that lncRNAs are differentially expressed in key pulmonary vascular cells such as PAECs and PASMCs, which regulate a range of cellular and biological processes and contribute to pulmonary vascular remodeling and PAH pathogenesis. Herein, we review the roles of lncRNAs in pathological processes in PAH (Figure 1 and Table 1).

### 2.1. LncRNAs and PASMC Dysfunction in PAH

Hyperproliferation and apoptosis resistance of PASMCs leads to pulmonary vascular remodeling which represents one of the key manifestations in PAH. The dysfunction of PASMCs is mediated by different signaling pathways, including PDGF, TGF-b, Wnt, hedgehog, estrogen, Notch, PI3K/AKT/mTOR and MAPK signaling [29]. Furthermore, apoptotic pathways, hypoxia, and non-coding RNAs, such as lncRNAs and miRNAs play key roles in PASMCs dysfunction [29]. In the following, we discuss the role of lncRNAs in PASMCs dysfunction and PAH pathogenesis.

#### 2.1.1. LncRNA H19

LncRNA H19 (referred to as H19 below) is a highly evolutionary conserved, imprinted maternally transcribed lncRNA, which is highly expressed during embryogenesis in extraembryonic tissues, in most fetal tissues, and in the embryo itself, but is downregulated postnatally, with the exception of the heart and skeletal muscle [53]. H19 has been reported to be associated in cancers with a range of cellular and physiological processes, including cell proliferation, differentiation, metabolism, hypoxia, inflammation, and oxidative stress [54,55,56]. Dysregulation of H19 has also been shown to be linked with other diseases, such as coronary artery disease [57], atherosclerosis [58], pulmonary fibrosis [59], and male infertility [60,61]. Recently, several studies revealed the regulatory role of H19 in the pathogenesis of PAH. H19 expression has been shown to be increased both in serum and lung of MCT-induced PH in rats and mice [32]. Additionally, H19 was shown to be induced by PDGF-BB, IL-1β, and IL-6 in PASMCs, and the PDGF-BB/H19/let-7b signaling axis promoted PASMC proliferation via regulating Angiotensin II receptor type 1, a novel target of the microRNA let-7b. Importantly, ablation of H19 in mice showed protection against MCT-induced PH features (RVSP, RV hypertrophy, pulmonary vessel medial wall thickness, and vessel muscularization), indicating that H19 could be a potential therapeutic target for PAH. However, in this study, the detailed mechanism of how PDGF regulates H19 remains unclear. Furthermore, additional studies are required to confirm these rodent model findings in human samples of PAH patients. In contrast to these findings, a recent study by Wang et al. demonstrated that MCT-induced PH rats have a significantly lower level of H19, PDCD4, and miR-675-3p, but higher expressions of IGF1R and miR-200a [62]. miR-200a and miR-675-3p targeted PDCD4 and IGF1R, respectively, and melatonin treatment modulated the H19/miR200a/PDCD4 and H19/miR-675-3p/IGF1R signaling axis, which inhibited proliferation but increased apoptosis in PASMCs. The role of H19 in pulmonary vascular remodeling is therefore unclear and controversially discussed.

#### 2.1.2. Tyrosine Kinase Receptor Inducing LncRNA (TYKRIL)

Using RNAseq data analysis of PASMC and lung pericytes from IPAH patients and hypoxia-exposed PASMC and pericytes, Zehendner et al., identified a novel lncRNA, called TYKRIL which is significantly increased in all four hyperproliferative conditions [30]. Under these hyperproliferative conditions, TYKRIL has been shown to promote proliferation and inhibit apoptosis in PASMC and pericytes by the p53/PDGF signaling axis [30]. Since TYKRIL has poor conservation in animals, the authors performed studies in ex vivo precision-cut lung slices (PCLS) collected from lungs of IPAH patients and demonstrated that GapmeR-mediated knockdown of TYKRIL in PCLS reverses pulmonary vascular remodeling [30], suggesting that TYKRIL has therapeutic potential for PAH treatment.

#### 2.1.3. Smooth Muscle Enriched Long Noncoding RNA (SMILR)

Expression of SMLIR has been shown to be highly expressed in PAH patients, in the MCT-induced PH model and hypoxia-induced human PASMCs (HPASMCs) [46]. Moreover, in vitro studies suggest that downregulation of SMILR inhibits hypoxia-induced proliferation and migration of PASMCs via targeting miR-141. Importantly, SMILR shRNA delivery in the MCT-induced PH rat model ameliorated PH and pulmonary vascular remodeling by targeting the Rho/ROCK/miR-141 axis [46], indicating that targeting SMILR has great potential for the treatment of PAH.

#### 2.1.4. PAXIP1-AS1

PAXIP1 antisense RNA 1 (PAXIP1-AS1) expression has been demonstrated to be upregulated in small pulmonary arteries, adventitial fibroblasts, and PASMCs of IPAH patients [37]. Mechanistically, PAXIP1-AS1 has been shown to affect focal adhesions by regulating their downstream target paxillin [37]. However, from this study, it remains unclear whether PAXIP1-AS1 plays a casual role in disease development or whether it is just a consequence of the remodeling processes.

#### 2.1.5. LncRNA Cancer Susceptibility Candidate 2 (CASC2)

LncRNA CASC2 (hereafter called CASC2) has been extensively investigated in a range of cancers where it regulates proliferation, migration, invasion, metastasis, and angiogenesis through different mechanisms. Only a few studies have shown the regulatory role of CASC2 in PAH. Gong et al. demonstrated that expression levels of CASC2 are significantly lower in PAs of hypoxia-induced PH in rats and HPASMCs [36]. Under hypoxic conditions, CASC2 overexpression was shown to inhibit cell proliferation and migration and increase apoptosis in both in vitro as well as in vivo. CASC2 overexpression also significantly reduced the expression levels of α-SMA, a phenotypic switch-related marker in hypoxia-induced PH in rats and HPASMCs. Importantly, CASC2 overexpression ameliorated hypoxia-induced experimental PH manifestations in rats, as evidenced by reduced mean pulmonary arterial pressure, RV hypertrophy, pulmonary vascular medial wall thickness, and fibrosis [36]. These findings indicate that CASC2 has functional involvement in PH at the molecular and cellular levels, as well as in vivo in experimental PH, however, further studies are needed to explore the underlying molecular mechanisms of how CASC2 suppresses the phenotypic switch and proliferation of PASMCs in hypoxia-induced PH. Another study demonstrated that CASC2 attenuated hypoxia-induced proliferation and migration of HPASMCs through modulating the miR-222/ inhibitor of growth 5 (ING5) signaling axis [63], suggesting that targeting CASC2 could be effective to suppress vascular remodeling in PAH.

#### 2.1.6. LncRNA Taurine-Up-Regulated Gene 1 (TUG1)

TUG1 is a 7.1-kb lncRNA located on chromosome 22q12.2 in the human genome. TUG1 plays a role in a variety of biological processes via different epigenetic mechanisms, including chromatin remodeling or by acting as decoys for micro-RNAs or proteins [64]. It has been shown that TUG1 acts as an oncogene or tumor suppressor in several types of cancers, including non-small cell lung cancer, colorectal cancer, and bladder cancer [65]. Recently, expression of TUG1 has been demonstrated to be upregulated in hypoxia-induced PH in mice and HPASMCs isolated from hypoxic mice. Elevated Tug1 enhanced the Foxc1 expression by sponging miR-374c, thereby stimulating proliferation and migration and inhibiting apoptosis in PASMCs to worsen pulmonary vascular remodeling through Notch signaling [38]. In line with these findings, Wang et al. also found the upregulation of TUG1 in the lungs of PAH patients and mice exposed to hypoxia and in hypoxic HPASMCs. Silencing of TUG1 protected against hypoxia-induced PH (RVSP, RV hypertrophy, and vessel wall thickness) in mice. Furthermore, silencing of TUG1, inhibited, while its overexpression promoted hypoxia-induced HPASMCs proliferation potentially via a miR-328 mediated mechanism [39]. These studies suggest that TUG1 plays a critical role in vascular remodeling and PAH with potential implications for PAH therapy. However, further studies are required to investigate whether TUG1 also modulate PAH through other mechanisms, for instance, inflammatory and immunological response, endothelial-to-mesenchymal transition, and phenotypic switch.

#### 2.1.7. LncRNA Antisense Noncoding RNA in the INK4 Locus (ANRIL)

ANRIL is a long noncoding RNA of 126 kbp containing 19 exons located at the INK4 locus of the chromosome 9p21 region [66]. Note that the INK4 locus is one of the strongest genetic susceptibility loci for cardiovascular diseases [67]. The lncRNA ANRIL was first discovered in patients with familial melanoma with neural system tumors. Prior studies suggest that ANRIL plays role in the pathogenesis of a range of diseases, such as coronary heart disease and atherosclerosis [67,68,69]. Dysregulation of ANRIL has been shown to be associated with dysfunction of vascular endothelium; the proliferation, apoptosis, migration, and senescence of VSMCs, mononuclear cell proliferation and adhesion, and DNA damage [67]. Moreover, siRNA-mediated silencing of ANRIL in human aortic VSMC suggested that splicing variants of ANRIL may participate in tissue remodeling [70]. These findings suggest that ANRIL may play a regulatory role in PAH pathogenesis. Recently, Wang et al. showed that ANRIL expression was significantly decreased in hypoxic HPASMCs [41]. Silencing of ANRIL with siRNA accelerated cell cycle progression and increased proliferation and migration of HPASMCs exposed to hypoxia [41], demonstrating that ANRIL played a crucial role in hypoxic HPASMCs with potentially far-reaching implications for the development of gene therapies and drugs for treating PAH. However, further studies are required to explore the underlying molecular mechanisms of how hypoxia-mediated downregulation of ANRIL contributes to the cellular functional alterations and pulmonary vascular remodeling in the pathogenesis of PAH.

#### 2.1.8. LncRNA Metastasis-Associated Lung Adenocarcinoma Transcript 1 (MALAT1)

MALAT1 is a widely expressed, highly conserved lncRNA of over 8 kb in length that is located on the human chromosome 11q13. It is one of the best-studied lncRNAs as it plays a role in the pathogenesis of a variety of cancers, including gastric, prostate, and non-small cell lung cancer. In a study of PAH patients and controls, Zhuo et al. identified a single nucleotide polymorphism (SNP) (rs619586A>G) in the MALAT1 gene which was linked with the susceptibility to PAH. The MALAT1 rs619586 GG allele presented a protective role on PAH. Mechanistically, it was proposed that the SNP genotype would directly enhance the levels of X box-binding protein 1 (XBP1) by competing for miR-214, and consequentially suppressing proliferation and migration of vascular endothelial cells in vitro by shortening S-M phase transition [71]. Moreover, MALAT1 SNPs were also investigated in a large cohort of children with and without congenital heart disease (CHD). The rs619586 GG allele was shown to be significantly linked with reduced CHD susceptibility possibly through upregulating the expression of MALAT1 [72]. Further studies are required to confirm whether MALAT1 plays a role in CTD-associated PAH. Wang et al. demonstrated that MALAT1 expression levels were significantly increased in the PAs and HPASMCs of PAH patients compared to the corresponding control samples. MALAT1 inhibition reduced cell cycle progression and proliferation, while overexpression had opposite effects. The MALAT1/hsa-miR-124-3p.1/KLF5 signaling axis was shown to enhance cell cycle progression as well as pulmonary vascular remodeling in PAH [43]. Another study showed that MALAT1 expression was increased in peripheral blood mononuclear cells (PBMCs) of PAH patients and hypoxia-induced HPASMCs. Further functional studies revealed that MALAT1 promoted HPASMCs migration and proliferation through regulating the miR-503/Toll-Like Receptor 4 signaling axis [44]. Intriguingly, hypoxia-induced MALAT1 expression was shown to be driven by HIF1a in HPASMCs. GapmeR-mediated silencing of MALAT1 reduced HPASMC proliferation and migration in vitro, and attenuated heart hypertrophy as evidenced by reduced relative weight of the RV/LV, while the RV pressure was unchanged in hypoxia-induced PH mice model [73]. However, this study neither showed evidence of its role in pulmonary vascular remodeling nor did it explore the role of the mechanisms of how MALAT1 exerted its effect on the RV.

#### 2.1.9. LincRNA-COX2

A recent study shows that the expression of lincRNA-COX2 was upregulated in peripheral blood samples from PAH patients and hypoxic PASMCs [48]. Further functional studies revealed that knockdown of lincRNA-COX2 reduced PASMCs proliferation via affecting the G2/M phase of the cell cycle under hypoxia. Knockdown of lincRNA also reduced PASMC migration under hypoxia. Importantly, lincRNA-COX2 was found to regulate the development of PAH via the STAT3/miR-let-7a signaling axis. These findings suggest a potentially important role of lincRNA-COX2 in PAH.

#### 2.1.10. LncRNA Ribosomal Protein S4-Like (RPS4L)

To identify lncRNAs related to PAH, Liu et al. performed a high throughput RNA-Seq analysis of PAs of hypoxic mice [49]. The authors identified nine upregulated and ten downregulated lncRNAs. Among the dysregulated lncRNAs, Rps4l was identified as a novel lncRNA that was downregulated in hypoxia-induced experimental PH in mice and hypoxic PASMCs. Overexpression of RPS4l in transgenic mice improved hypoxia-induced experimental PH features, as evidenced by improved RVSP, RV hypertrophy, cardiac function, hemodynamics, and pulmonary vascular remodeling. Consistent with these in vivo findings, lentiviral vector mediated-overexpression of RPS4L inhibited the proliferation and attenuated cell cycle progression triggered by hypoxia in mouse PASMCs. Exploring the underlying molecular mechanism, the authors demonstrated that RPS4L expression was reduced in PASMCs due to hypoxia and that decreased RPS4L levels regulated proliferation, migration, and cell cycle progression via interleukin enhancer-binding factor 3 (ILF3)/HIF-1α signaling pathways. Although, these findings may offer new insight into the pathomechanisms of hypoxia induced PH, thereby providing an important basis for discovering new treatments, these findings need validation in tissues and cells of PAH patients. In addition, this study used the hypoxic PH mouse model, which is not a perfect and comprehensive model to mimic all the human PAH features. Further studies are required to perform experiments in different other animal models such as Sugen5416-hypoxia, and monocrotaline models, which more closely mimic human PAH. In a different study, the same research team identified a RPS4L-encoding peptide, called RPS4XL (RPS4 X isoform-like), which regulates RPS6 phosphorylation and reduced PASMCs proliferation triggered by hypoxia [74], suggesting that this peptide could be effective in hypoxic PH. However, again these in vitro findings are required to be confirmed in vivo and in samples of patients with PAH.

#### 2.1.11. LncRNA Pulmonary Arterial Hypertension Related Factor (PAHRF)

LncRNA PAHRF, also known as NONHSAT169231.1, is located on chromosome 14 in the human genome. HPASMCs exposed to hypoxia as well as PAs from patients with PAH were found to have low PAHRF expression compared to the corresponding control samples [51]. PAHRF overexpression inhibited proliferation and promoted apoptosis in HPASMCs, whereas knockdown resulted in opposite effects [51]. Further mechanistic studies suggest that downregulation of PAHRF expression serves as a miR-23a-3p sponge to promote pulmonary vascular remodeling in hypoxic pulmonary hypertension by stimulating the proliferation of HPASMCs and inhibiting their apoptosis by downregulating MST1 expression [51]. This study suggests that PAHRF/miR-23a-3p/ MST1 signaling axis could be a possible therapeutic target for PAH treatments by improving pulmonary vascular remodeling.

#### 2.1.12. LncRNA Growth Arrest-Specific Transcript 5 (GAS5)

GAS5 plays a crucial role in cell growth arrest and apoptosis [75]. Expression of GAS5 has been shown to be downregulated in a variety of cancers, such as lung, breast, bladder, gastric, prostate, and pancreatic cancer [75], suggesting that GAS5 may play a tumor suppressor role during cancer progression. GAS5 modulates TGF-beta-induced SMC differentiation through RNA Smad-binding elements [76]. Additionally, GAS5 regulates SMC function possibly via β-catenin signaling [77]. Recently, in the lung of hypoxia-induced PH rats and hypoxic PASMCs, GAS5 expression was found to be significantly downregulated [52]. Furthermore, silencing of GAS5 expression with siRNA stimulated migration and proliferation of HPASMCs [52]. Mechanistically, GAS5 regulates migration and proliferation of HPASMCs potentially via Gas5/miR-23b-3p/KCNK3 signaling axis [52].

### 2.2. LncRNAs and Endothelial Cell Dysfunction in PAH

Abnormal proliferation and angiogenesis of PAECs are important contributors to the development of PAH. LncRNAs can directly or indirectly play a regulatory role in endothelial cell dysfunction.

#### 2.2.1. LncRNA MANTIS

LncRNA MANTIS (also known as n342419) expression was shown to be reduced in the lungs of MCT-induced PH rat models as well as end-stage patients with IPAH [40]. Conversely, a significantly higher level of MANTIS was found in ECs isolated from human glioblastoma patients and in the carotid arteries of Macaca fascicularis fed an atherosclerosis diet with a subsequent regression phase [40]. MANTIS was found to be controlled by the histone demethylase JARID1B and localized in the nucleus, suggesting that MANTIS may play role in chromatin regulation. MANTIS deletion with CRISPR/Cas9 or knockdown with GapmeRs or siRNAs reduced EC migration, tube formation, angiogenic sprouting in vitro as well as in vivo in mice injected with matrigel-embedded human umbilical vein endothelial cells (HUVECs). Further mechanistic studies revealed that MANTIS binds to BRG1 and controls angiogenesis-regulating genes SOX18, COUP-TFII, and SMAD6 [40].

#### 2.2.2. LncRNA MALAT1

An earlier report by Lin et al. revealed that human MALAT1 can act as a global modulator of RNA post-transcriptional modification in HUVECs [78], pointing to a potential role of MALAT1 in endothelial dysfunction. This was validated by the fact of hypoxia-induced MALAT1 expression in HUVECs in vitro, and of genetic deletion or inhibition of MALAT1 impaired cell proliferation and vascularization in vivo [79]. Furthermore, downregulation of miR-126 expression was found to be negatively linked with upregulated MALAT-1, which contributed to the impairment of angiogenesis in the RV of PAH patients, and reduced tube formation and ECs proliferation in isolated ECs from the RV of PAH patients.

### 2.3. LncRNAs and Growth Factors Signaling in PAH

An imbalance in PDGF signaling may result in dysregulation of PASMC proliferation, thereby causing vascular remodeling in PAH. Mechanistically, PDGF strongly promotes SMC proliferation by activating PDGFR tyrosine kinases and downstream signaling pathways effectors, such as phosphatidylinositol 3-kinase (PI3K), Src family kinases, Src-homology 2 domain-containing protein tyrosine phosphatase, and phospholipase C γ [80]. Levels of PDGF and PDGFRs are increased in the lungs of PAH patients and in experimental murine models of PH [81,82]. Importantly, PDGF inhibition with imatinib was shown to reverse PAH features in MCT-induced rats and chronic hypoxia-treated mice [83]. To investigate the role of lncRNAs in orchestrating PDGF signaling in the pathogenesis of PAH, Chen and colleagues identified 39 upregulated and 56 downregulated lncRNAs in a RNAseq data set of PDGF-stimulated rat PASMCs. Based on the highest expression and species conservation, the authors focused on the lncRNA regulated by PDGF and transforming growth factor β (LnRPT) and identified a signaling axis PDGF-PI3K-LnRPT-NOTCH3 which regulated PASMC proliferation [50]. These findings may help understand the complex mechanisms involved in regulating PASMC proliferation and may be useful in designing new therapeutic targets to inhibit PASMCs proliferation.

### 2.4. LncRNAs and Metabolic Dysfunction in PAH

Metabolic dysregulation is considered as one of the key hallmark features of PAH. Mitochondria are the crucial regulators of the complex metabolic pathways. In patients with PAH, pulmonary vascular remodeling has been shown to be linked with a large number of aberrant metabolic pathways, such as changes in glucose and fatty acid oxidation and glycolysis, glutaminolysis, one-carbon metabolism, arginine metabolism, the tricarboxylic acid cycle, and the oxidizing and reducing cell environment [84]. A growing body of evidence suggests that lncRNAs are directly or indirectly involved in mitochondrial biology in health and disease [85]. For example, miRNA processing-related lncRNA and mitochondrial dynamic related lncRNA regulate mitochondrial dynamics through targeting fission 1 expression [86]. LncRNA SAMSON regulates mitochondrial precursor-over-accumulation stress, membrane potential depolarization, and cell apoptosis in melanoma via targeting p32 [87]. LncRNA RMRP stimulates mitochondrial respiration in Hela cells [88]. LncRNA Tug1 controls the activity of respiratory complex I and III and increases mitochondrial bioenergetics through interacting with tug1-binding element upstream of peroxisome proliferator-activated receptor-c coactivator-1α [89]. LncRNA H19 stimulates melanoma cell glucose metabolism through miR-106a-5p/E2F3 [55]. LncRNA UCA1 indirectly promotes glycolysis in bladder cancer cells via mTOR-STAT3/miR143/hexokinase 2 axis [90]. H19 and MALAT1 promote hepatic lipogenesis via stabilizing the sterol regulatory element-binding protein-1c protein [91,92]. MALAT1 also regulates glucose metabolism, increases glycolysis, and inhibits gluconeogenesis through a higher level of translation of the transcription factor TCF7L2, which contributes to the development of hepatocellular carcinoma [93]. LncRNA ANRIL promotes development of acute myeloid leukemia via a glucose metabolism pathway of AdipoR1/AMPK/SIRT1 [94]. Importantly, as described above, MALAT1, H19, TUG1, and ANRIL are also implicated in pulmonary vascular remodeling and PAH, however, whether the role they play in metabolic dysfunction in the disease is yet to be explored.

### 2.5. LncRNAs and RV Remodeling in PAH

In PAH, the RV endures complex remodeling, including RV pressure overload, changes in myocardial extracellular matrix, and an increase in cardiomyocyte size, and enhanced capillary angiogenesis and intrinsic contractility [95]. As a result of prolonged pressure overload, adaptive response gradually changes into maladaptive remodeling and eventually leads to RV failure and death. Even though different regulatory mechanisms and stimuli of RV remodeling in PAH have been identified, there is still no known cure for the disease. Currently available treatments are typically focused on symptom relief and delaying the disease progression. Thus, there is an urgent need to find new targets that can improve RV remodeling during PAH. Recently, lncRNAs have been shown to be differentially expressed in the RV of patients with PAH and or animal models [31,96]. Further detailed studies suggest that lncRNA H19 plays an important role in RV remodeling. Increased cardiac expression of H19 is more consistently linked to heart failure. H19 has been consistently found to be expressed at higher levels in the failing left ventricle (LV) from both human and animal models [97,98]. H19 has also been shown to stimulate cardiac fibroblast proliferation [99], promote cardiomyocyte apoptosis in dilated cardiomyopathy [100], and modulate cardiomyocyte hypertrophy [101], all relevant to the progression of RV failure. In a recent study by Omura et al., H19 expression was shown to be increased in the decompensated RV of PAH patients, which positively correlated with RV hypertrophy and fibrosis. Consistent with these clinical findings, H19 was overexpressed in decompensated RV of two PH rat models (PAB and MCT) [31]. Furthermore, GapmeR-mediated downregulation of H19 in PAB- and MCT-induced PH rat models improved PAH features, including RV hypertrophy, capillary rarefaction, and fibrosis without influencing pulmonary vascular remodeling [31]. These cardioprotective effects were linked with E2F transcription factor 1-mediated up-regulation of the zesty homolog enhancer. Silencing of H19 inhibited phenylephrine-induced cardiomyocyte hypertrophy while its overexpression reversed the effect [31]. It has also been found that elevated levels of H19 in plasma from patients with PAH are predictive of long-term survival, discriminate them from controls, and correlate with RV function [31]. Together these findings suggest that targeting H19 has the potential to be a promising therapeutic option for PAH-related RV failure and circulatory levels of H19 could be used as a potential biomarker for PAH prognosis and severity.

## 3. LncRNAs as a Potential Biomarkers in PAH

LncRNAs could be used as a potential biomarkers in PAH. For instance, in a study of 736 healthy controls and 587 Chinese PAH patients, a functional polymorphism of lncRNA MALAT1 (rs619586A>G) has been shown to be linked with a decreased susceptibility to PAH. Mechanistically, this polymorphism in lncRNA MALATI created a miRNA sponge site for miR-214 and thereby increased the expression of X box binding protein 1 (XBP1) expression [71]. As another example, qPCR analysis of plasma levels of lncRNA H19 expressions were shown to be higher in IPAH patients in two cohorts of PAH patients and controls from Canada (52 IPAH and 57 controls) and the UK (75 IPAH and 54 controls). The circulatory H19 levels, partially discriminating PAH patients from controls, were modestly linked with RV function in PAH patients, and were predominantly increased in PAH patients with a low cardiac index. Finally, higher levels of H19 were associated with poorer long-term outcomes in both cohorts. In contrast, a previous study found no difference in the expression of H19 in plasma between eight PAH patients and eight healthy controls, as assessed by qPCR [102]. It is notable that the authors of this study did not specify which PAH types were used (e.g., idiopathic, heritable, or connective tissue diseases associated etc.). In this relatively small sample size analysis, low abundance of H19 in plasma may cause high variability that could potentially render the results statistically insignificant. Further studies are needed to confirm these findings in a larger cohort of patients with different PAH types. Taken together, these findings suggest a potential biomarker role of lncRNAs in PAH.

## 4. Challenges of lncRNAs Research in PAH

A large number of lncRNAs have been identified in samples of PAH patients and preclinical models using next generation sequencing tools, especially RNAseq and other methods. However, the lncRNAs are poorly conserved across species in terms of their functions, structures, and sequences; thus, it is challenging to study these lncRNA in vivo in preclinical animal models of PH required for clinical translation, as some lncRNAs are expressed in humans only. In addition, accurate measurement, and detection of lncRNAs in the circulation and tissues is challenging due to their low abundancy, possible degradation, and potential mixing with cell, cell debris, and platelets. Highly sensitive RNA detection techniques and deeper RNA sequencing are needed to reveal the possible roles of lncRNAs in health and disease.

## 5. Therapeutic Potential of lncRNAs in PAH

LncRNAs play a critical role in the pathogenesis of PAH; therefore, a deeper understanding of their function and mechanism of action is of utmost importance to develop novel and effective therapies for this disease. Since expression of lncRNAs are highly cell/tissue-specific, they could be used as a potential tools for personalized treatments. Injection of BC-819, a double-stranded DNA plasmid containing a gene for diphtheria toxin under the regulation of the H19 gene promoter into a mouse bladder tumor, for example, decreased tumor size [103]. Combined treatment of BC-819 and Bacilli Calmette Guerin (BCG) was shown to significantly improve patients’ outcomes with a recurrence free bladder cancer survival rate of 54.1% and a progression free survival rate of 75.7% over 24-months in a Phase II clinical trial in patients with non-muscle invasive bladder cancer, suggesting that lncRNA-based treatments have a promising future.

To date, no clinical trials targeting lncRNAs in PAH have been carried out as many obstacles remain. Foremost, lncRNAs are poorly conserved across species, which makes it difficult to develop and test new drugs for clinical translation of human lncRNAs. Also, several lncRNAs have shown promising therapeutic potential in in vitro and in vivo PAH studies, however, in clinical studies the use of lncRNA therapy will need to be handled with caution due to the potential effect on multiple cellular pathways in different cell types and tissues. Antisense oligonucleotides, RNA interference drugs, and CRISPR genome editing tools can be used to alter lncRNA expression as a potential therapeutic option for treating PAH. Furthermore, before a successful translation of preclinical lncRNA studies into clinical use, other issues must be resolved such as improving the stability of RNA-based drugs in the circulation, identifying the secondary structure of lncRNAs, determining the proper route of delivery, improving the efficiency and duration of delivery systems, determining the speed of onset and duration of their action, and limiting off-target effects and adjusting patient-specific doses.

## 6. Concluding Remarks and Future Perspectives

LncRNAs play critical roles in a number of biological processes involved in pulmonary vascular remodeling and PAH. Moreover, misregulated lncRNAs may lead to cellular dysfunctions, such as cell proliferation, apoptosis, migration, angiogenesis, metabolism, regulation of the phenotypic switching, and cell cycle, which might contribute to the development and progression of the disease. Although the precise role or function and mechanism of action of lncRNAs in the onset and progression of PAH largely remains unknown. Most of the studies conducted in the field, focused on pre-selected lncRNAs rather than identifying the specific lncRNA that was most important in the disease pathogenesis. One of the main functions of lncRNAs within the cell is competition with miRNAs to bind mRNA targets. Binding sites for more than one miRNA may exist in the same lncRNA, but most studies are focused only on a single lncRNA-single miRNA interaction rather than looking at global effects of the lncRNAs. New tools such as high-throughput screening of protein, RNA and DNA-binding chaperones of lncRNAs, high resolution imaging of lncRNAs, novel CRISPR-Cas9 mediated intervention in vivo as well as in vitro, currently available lncRNA databases, and potentially a PAH-lncRNAs-specific bioinformatics database could help to better understand key molecular mechanisms underlying the pathogenesis of PAH (Table 2). Finally, to advance PAH treatment, it would be beneficial to leverage the diagnostic, therapeutic, and prognostic biomarker potential of lncRNAs both in animal models and humans. Although, this goal may seem elusive, it can be obtained by accelerating translational research.

## Figures and Tables

**Table 1:** Summary of lncRNAs involved in PAH.

LncRNA	Subcellular Localization	Experimental Models Used	Expression in PAH	Key Findings	Mechanisms	Ref
TYKRIL	Nucleus and cytoplasm	Human PAH PCLS. Hypoxia-induced PH in HPASMCs	↑ in PASMC, pericytes	Enhances proliferation, inhibits apoptosis of HPASMCs under hypoxia. Regulates pulmonary vascular remodeling in PCLS.	p53/PDGFRβ axis	[30]
H19	-	MCT-, and PAB-induced PH rat models	↑ in decompensated RV of PAH patients	Correlates with fibrosis and RV hypertrophy in PAH patients. Compromises the RV functions in PAH, inhibition showed benefits in two PH animal models. Plasma levels of H19 showed biomarker potentials in two independent IPAH cohorts	EZH2	[31]
H19	-	MCT-induced PH mouse and rat model	↑ in serum, lung	Increases PDGF-induced PASMC proliferation. Depletion of H19 protects against MCT-induced PA remodeling and PAH in mice	miRNA let-7b/ AT1R	[32]
MEG3	Predominantly in cytoplasm	Hypoxia-induced PH in HPASMCs	↓ in lung, PA, PASMC	Inhibits HPASMCs proliferation and migration	miR-21/PTEN; p53 pathway	[33,34]
MEG3	Predominantly in cytoplasm	Hypoxia-induced PH in mice and HPASMCs	↑ in PASMC	Downregulation inhibits hypoxia-induced PH development in mice, Hyperactivates cell cycle progression, increases proliferation of PASMC under hypoxia.	miR-328-3p/IGF1R	[35]
CASC2	-	Hypoxia-induced PH in rats and PASMCs	↓ in PA, PASMC	Inhibits hypoxia-induced vascular remodeling in vivo, inhibits cell proliferation, migration, and phenotypic switch of PASMC	α-SMA	[36]
PAXIP1-AS1	Both nucleus and cytoplasm	Small PAs of IPAH patients	↑ IPAH-PASMCs and PAs	Inhibition promotes apoptosis and inhibits PASMC proliferation and migration	Paxillin	[37]
TUG1	Both nucleus and cytoplasm	Hypoxia-induced PH in mice and HPASMCs	↑ in PA	Inhibition prevents and inhibits PH in hypoxia-induced PH mouse models. Promotes HPASMC proliferation and cell cycle progression in vitro	miR-374c/Foxc1, notch signalling	[38,39]
MANTIS	Nucleus	MCT-induced PH rat models	↓ in lung	Promotes apoptosis, accelerates endothelial angiogenic function.	BRG1	[40]
ANRIL	-	Hypoxia-induced PH in HPASMCs	↓ in PASMC	Promotes proliferation and migration of PASMC	Unknown	[41]
HOXA-AS3	-	Hypoxia-induced PH in HPASMCs	↑ in lung, PASMC	Modulates cell cycle and enhance proliferation in PASMC	HOXA3	[42]
MALAT1	-	-	↑ in PA, PASMC	Accelerates cell cycle progression as well as pulmonary vascular remodeling	hsa-miR-124-3p.1/KLF5	[43]
MALAT1	-	Hypoxia-induced PH in HPASMCs	↑ in plasma and hypoxic HPASMCs	Knockdown reduces HPASMCs proliferation and migration while promotes their apoptosis	miR-503/TLR4 Axis	[44]
UCA1	Predominantly in cytoplasm	Hypoxia-induced PH in HPASMCs	↑ in PASMC	Promotes proliferation and inhibits apoptosis in PASMC	hnRNP I	[45]
SMILR	-	Hypoxia-induced PH in HPASMCs, MCT-induced PH in Rats	↑ in serum	Regulates vascular remodeling and PAH	RhoA/ROCK/miR-141 signaling	[46]
TCONS_00034812	-	Hypoxia-induced PH in Rats and PASMCs	↓ in PA, PASMC	Promotes proliferation and inhibits apoptosis of PASMC	Stox1/MAPK signaling	[47]
LincRNA-COX2	-	Hypoxia-induced PH in PASMCs	↑ in blood, PASMCs	Promotes PASMCs proliferation	LincRNA-COX2/miR-let-7a/STAT3 axis	[48]
RPS4L	-	Hypoxia-induced PH in mice and HPASMCs	↓ in PASMC	Ameliorates hypoxia-induced PH in vivo. Modulates proliferation, migration, and cell cycle progression of PASMC	ILF3/HIF1α	[49]
LnRPT	-	PDGF-BB-induced hyperproliferation of rat PASMCs	↓ in PASMC	Promotes PASMC proliferation	Notch signaling pathway	[50]
PAHRF	-	Hypoxia-induced PAH in PASMC	↓ in PAs of PAH patients and hypoxic PASMC and	Downregulation of PAHRF promotes PASMC proliferation and inhibits apoptosis	PAHR/ miR-23a-3p/MST1 axis	[51]
GAS5	-	Hypoxia-induced PH in rats and PASMC	↓ in rats PH models and hypoxic PASMC	Downregulation of Gas5 promotes PASMC proliferation	Gas5/miR-23b-3p/KCNK3 axis	[52]

**Table 2:** List of tools to better understand key molecular mechanisms underlying the pathogenesis of PAH.

Tools	Application	Ref
Crosslinked immunoprecipitation	lncRNA-protein interaction	[104]
RNA antisense purification	lncRNA-protein interaction	[105]
Chromatin isolation by RNA purification	lncRNA-protein interaction	[106]
Capture hybridization analysis of RNA targets	lncRNA-protein interaction	[107]
Selective 2′-hydroxyl acylation analyzed by primer extension sequencing	lncRNA structural information	[108]
Dimethyl sulfate sequencing	Structural changes	[109]
CRISPR/Cas9 (CRISPR activation and CRISPR interference)	lncRNA function	[30]
High resolution RNA imaging techniques	Visualizing RNA expression, localization	-
LncRNA-databases such as LncRNAdb v2.0, LncRNA2Target, lncRNAMap, ENCODE, FANTOM, LNCipedia 3.0, lnCeDB, DIANA TOOLS, LncBase v.2, NRED	lncRNAs information	[110]
PAH-specific lncRNAs database	Needs to develop to share lncRNAs RNAseq data, structure-functional analysis data, structure-function prediction analysis, etc.	-
PAH biobanks, such as the UK PAH Cohort Study Consortium and the US PAH Biobank Consortium	Large collections of existing PAH patient biospecimens, including plasma, could help future biomarker research	-
